# Supplementary material for: Drug repurposing for aging research using model organisms
Source: Aging Cell. 2017 Jun 16;16(5):1006–15. doi: 10.1111/acel.12626 (PMC5595691; doi:10.1111/acel.12626)
Supplement: Supplementary file 7 — Data S1 Zip‐Archive of all report cards. [file ACEL-16-1006-s007.zip › RC_AKG.pdf]

AKG

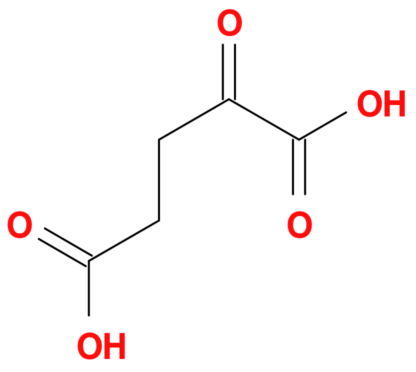

Database identifiers

ChEMBLCompound	CHEMBL1686
DrugBank	DB02926
CHEBI	30915
eMolecules	530280

Ranking

	Rank	Score
Drosophila	NA	NA
C. elegans	240/591	0.225

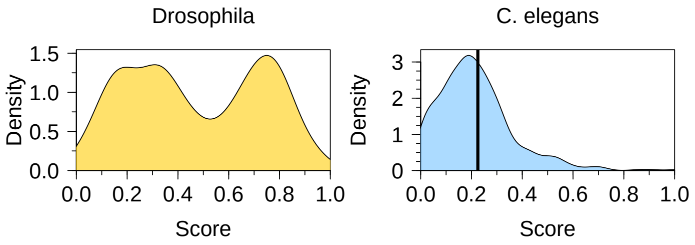

	Ageing implication	Domain conservation	Binding site conservation	Binding affinity	Bioavailability	Lipinski	Promiscuity	Purchasability	Drug approval	**Total**
Drosophila	NA	NA	NA	NA	NA	NA	NA	NA	NA	NA
C. elegans	0.81	0.982	1.0	0.292	0.218	0.0	-0.0	0.1	0.075	0.225

Names

- **2-oxoglutaric acid**
- 2-Ketoglutaric acid
- 2-Oxoglutarate
- 2-Oxopentanedioic Acid
- Alpha-Ketoglutarate
- alpha-ketoglutaric acid

Roles

fundamental metabolite

Status

Approved drug (according to ChEMBL)	No
Number of Rule of 5 violations	0
Binding affinity to original target in log units (RF-Score prediction)	4.11
Burns *C. elegans* bioavailability prediction	-4.48

Compound Target Characteristics

Lysine-specific demethylase 7 homolog

Best gene implication in ageing for this target family came from gene Q9GYI0 via mapping the annotation from WormBase WBGene00017920 annotated in WormBase 2014-03-11. Annotation GO 8340 (determination of adult lifespan) was Inferred from Mutant Phenotype

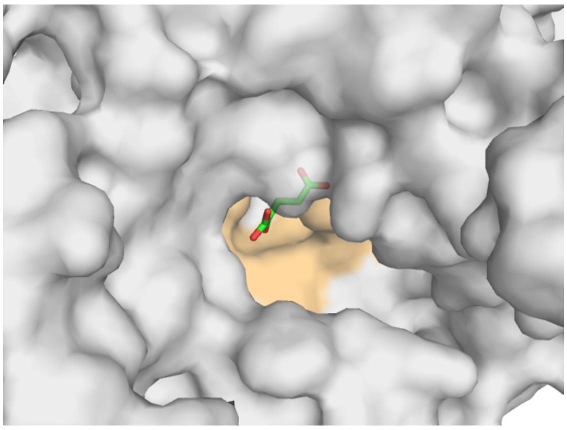

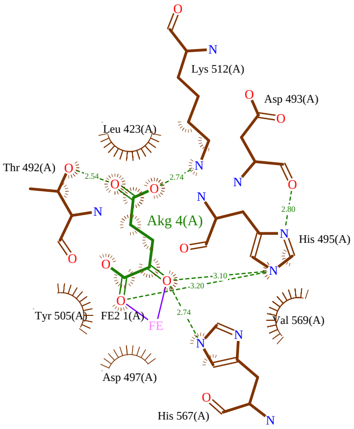

protein	amino acids contacts (binding site)
PDB:3puq:chainA:Q9GYI0	N L T H D Y K H V
sp:Q9GYI0:KDM7_CAEEL	N L T H D Y K H V

	whole protein		domain-based		contact-based	
protein	ident	simil	ident	simil	ident	simil
PDB:3puq:chainA:Q9GYI0	1.0	1.0	1.0	1.0	1.0	1.0
sp:Q9GYI0:KDM7_CAEEL	1.0	1.0	1.0	1.0	1.0	1.0

jmjd-1.2 (UniProt:Q9GYI0) annotation

Function: Histone demethylase required for nervous system development. Specifically demethylates dimethylated 'Lys-9' and 'Lys-27' (H3K9me2 and H3K27me2, respectively) of histone H3, thereby playing a central role in histone code. (PubMed:20346720, PubMed:20567261, PubMed:20567262).
Cofactor: Fe(2+) Evidence=(PubMed:20567261); Note=Binds 1 Fe(2+) ion per subunit. (PubMed:20567261);
Enzyme regulation: Competitively inhibited by 2-hydroxyglutarate. (PubMed:21251613).
Subcellular location: Nucleus (PubMed:20346720).
Tissue specificity: Mainly expressed in neurons. Also weakly expressed in some muscle, intestinal and hypodermal cells. (PubMed:20346720).
Domain: The PHD-type zinc finger mediates binding to H3K4me3. (PubMed:20567261, PubMed:20567262).
Disruption phenotype: Impaired locomotion. While wild-type animals move by alternating dorsal and ventral flexions along the body length, producing a regular sinusoidal track on bacteria, the track pattern left mutants is irregular, with increased wavelength (distance between successive peaks of a wave) but unchanged amplitude of the wave. An increase of H3K9me2 and H3K27me2 levels is observed. (PubMed:20346720).

(Information from UniProt)
